# Characterization of Food Structures and Functionalities

**DOI:** 10.1155/2018/4818253

**Published:** 2018-04-19

**Authors:** Xingxun Liu, Ying Yang, Changmou Xu, Jiajia Rao

**Affiliations:** ^1^Institute of Food Science and Technology (IFST), Chinese Academy of Agricultural Science (CAAS), Beijing, China; ^2^Food Science and Technology Department, University of Nebraska–Lincoln, Lincoln, NE, USA; ^3^Department of Plant Sciences, North Dakota State University, Fargo, ND, USA

Some functional properties such as texture and nutrition are the most important attributes used by consumers to assess food qualities, which have been used for in nearly all kinds of food products, from beverage, yoghurt, and ice cream to bread and noodles. Nowadays, there is a desire to make foods healthier and at the same time not diminish sensory quality. This requires an understanding of key elements of food structure associated with texture and nutritional perception. In terms of taste perception, texture is perceived during oral processing of food.

Knowledge of structure-oral processing-texture interrelations could be utilized to develop or prevent specified textural attributes. Overall, the investigation of structure-oral processing-texture interrelations is just starting as a research focus preferentially. However, factors are as including nonuniversal and inconsistent sensory terminology, omission of consideration for structural changes incurred by oral processes, the imbalances between texture and nutrition occurring during food design, the confusions of the key elements of food structure to determine food texture or nutrition, and the lack of cross-disciplinary investigations hamper progress in this field. Consideration of these factors in future investigations on sensory texture and nutrition functionalities, as well as the development of the relevant analytical methods for studying the structural changes or evaluating texture and nutrition functionality changes, will increase the applicability of their findings and bring us closer to understanding the contribution of food structure to sensory texture while helping us to make a balance between food textures and food functionalities during food design.

Nowadays varieties of analysis methods based on spectral analysis and chromatographic analysis have been developed to identify and analyze food composition and food structure more quickly. Technologies such as dynamic light scattering, X-ray, neutron scattering, and various microscopy (optical and electron) techniques were applied to study the food structure from different scale level, while some other analysis technologies have been developed to mimic the oral chewing procedure and the digestion procedure for food textures and food functionalities study. All these developments will help us get a better overall understanding of the relationship between food structures and functionalities.

In recent years, varieties of analysis methods were used to characterize food structures and functionalities and have experienced a rapid development with the purpose of improving food systems. This special issue aims to provide an opportunity for researchers in the area of characterization of food structures and functionalities to share their state-of-the-art researches. These original researches uncovered the development of food characterization and specifically include the following: (i) advanced technologies of analyzing the structures of food; (ii) advanced technologies of analyzing the textures of food; (iii) advanced technologies of analyzing the nutrition and functionalities of food; (iv) investigation of the interrelations between food compositions/food structures and food textures/nutrition; (v) analytical model from food structures to food textures/nutrition.

We hope readers will benefit for the researches included in this special issue.

